# An AI-Powered Clinical Decision Support System to Predict Flares in Rheumatoid Arthritis: A Pilot Study

**DOI:** 10.3390/diagnostics13010148

**Published:** 2023-01-01

**Authors:** Hannah Labinsky, Dubravka Ukalovic, Fabian Hartmann, Vanessa Runft, André Wichmann, Jan Jakubcik, Kira Gambel, Katharina Otani, Harriet Morf, Jule Taubmann, Filippo Fagni, Arnd Kleyer, David Simon, Georg Schett, Matthias Reichert, Johannes Knitza

**Affiliations:** 1 Department of Internal Medicine 3-Rheumatology and Immunology, Friedrich-Alexander University Erlangen-Nürnberg and Universitätsklinikum Erlangen, 91054 Erlangen, Germany; 2 Deutsches Zentrum für Immuntherapie, Friedrich-Alexander University Erlangen-Nürnberg and Universitätsklinikum Erlangen, 91054 Erlangen, Germany; 3 Siemens Healthineers, 91502 Erlangen, Germany

**Keywords:** artificial intelligence, machine learning, rheumatoid arthritis, flare prediction, clinical decision support system, CDSS, eHealth, digital health

## Abstract

Treat-to-target (T2T) is a main therapeutic strategy in rheumatology; however, patients and rheumatologists currently have little support in making the best treatment decision. Clinical decision support systems (CDSSs) could offer this support. The aim of this study was to investigate the accuracy, effectiveness, usability, and acceptance of such a CDSS—Rheuma Care Manager (RCM)—including an artificial intelligence (AI)-powered flare risk prediction tool to support the management of rheumatoid arthritis (RA). Longitudinal clinical routine data of RA patients were used to develop and test the RCM. Based on ten real-world patient vignettes, five physicians were asked to assess patients’ flare risk, provide a treatment decision, and assess their decision confidence without and with access to the RCM for predicting flare risk. RCM usability and acceptance were assessed using the system usability scale (SUS) and net promoter score (NPS). The flare prediction tool reached a sensitivity of 72%, a specificity of 76%, and an AUROC of 0.80. Perceived flare risk and treatment decisions varied largely between physicians. Having access to the flare risk prediction feature numerically increased decision confidence (3.5/5 to 3.7/5), reduced deviations between physicians and the prediction tool (20% to 12% for half dosage flare prediction), and resulted in more treatment reductions (42% to 50% vs. 20%). RCM usability (SUS) was rated as good (82/100) and was well accepted (mean NPS score 7/10). CDSS usage could support physicians by decreasing assessment deviations and increasing treatment decision confidence.

## 1. Introduction

RA is a chronic inflammatory disease that leads to joint damage and bone destruction, causing severe pain, disability, and reduced life expectancy [1,2,3]. Treat-to-target (T2T) with disease-modifying antirheumatic drugs (DMARDs) has become the gold standard of care for RA patients [4,5]. The T2T concept, which advises escalating therapy in RA patients with moderate and high disease activity, helps to achieve fast disease control and to prevent structural damage and functional limitations in patients with RA. Once RA patients reach remission [6], tapering, i.e., a gradual reduction in the dose of the drug, may be feasible. Current guidelines indicate tapering DMARDs in patients in persistent remission for at least 6 months [4,5]. Tapering may minimize side effects and reduce drug burden. Moreover, health system-related savings and the fairer distribution of resources can be achieved [3]. On the other hand, tapering leads to the increased occurrence of flares in some patients.

In clinical practice, patients and rheumatologists currently have little support in making the best treatment decision. Various studies have suggested predictive factors for RA flares that should be considered, such as remission duration, anti-citrullinated protein antibody (anti-CCP) status, and multi-biomarker disease activity (MBDA) score [7,8,9]. However, the availability, collection, and weighting of different factors complicate the treatment decision making of patients and rheumatologists, and even experts may be inconsistent in judgments and perform worse than algorithms [10]. Furthermore, decisions based on subjective experiences lead to heterogeneous decisions between rheumatologists. In addition, increasing time and performance pressure on health care professionals’ prompt heuristic thinking, which may foster clinical mistakes [11].

Clinical decision support systems (CDSSs) may offer solutions to these challenges. CDSSs have been shown to improve clinical practice, medication dosing, preventive care, and other care aspects in a wide range of medical disciplines [12,13], and applications using artificial intelligence (AI) have been applied to predict disease and mortality for various clinical conditions [14,15,16,17]. Recently, we developed a flare prediction tool based on machine learning (ML) for RA patients [18].

The aim of this study was to investigate the accuracy, effectiveness, usability, and physician acceptance of an AI-powered flare prediction RCM to support the management of RA.

## 2. Materials and Methods

### 2.1. Rheuma Care Manager (RCM) including Flare Prediction Tool

The RCM, a CDDS, was used as the software prototype version 1.0.74. The RCM consists of two parts: (i) a floating patient overview and (ii) a flare risk prediction tool. The patient overview displays the patient’s history (previous and current medication, age, sex, body mass index (BMI), smoker status, disease duration, comorbidities, anti-CCP status, disease status, last CRP (C-reactive protein) and ESR (erythrocyte sedimentation rate) values, and a visual timeline including DAS28-ESR (disease activity score, 28 joints, erythrocyte sedimentation rate) disease activity, and medication). The prediction tool displays the predicted risks of a disease flare for the RA patient in sustained remission within a period of 14 weeks as percentage bar graphs for two scenarios: the continuation of current medication vs. a half dosage of current medication.

The flare risk prediction tool is a machine learning model that was developed based on data from clinical routines (73 RA patients) and RETRO studies (40 patients) [19]. The RETRO data were synthetically oversampled to increase the tapering rate in the final training data set (258 patients, 557 visits), which increased the learning ability. The model’s risk prediction uses 10 clinical variables: DAS28-ESR; disease duration; administration form of biologic DMARD (bDMARD, intravenous, not intravenous); anti-CCP (positive or negative); gender; HAQ (health assessment questionnaire); CRP; bDMARD dose (half, full); swollen joint count (SJC); and tender joint count (TJC). Additionally, users are presented with the underlying impact of each variable for the risk prediction, which was determined using SHAP (Shapley additive explanations) to make the AI-based prediction explainable [20,21]. SHAP is a game-theoretic approach [20]. The SHAP values reflect each variable’s contribution to the individual risk and if the variable is a risk-increasing or -decreasing factor. Relative importance was calculated by dividing the predictor’s flare risk contribution by the sum of the total predictors’ flare risk contributions.

### 2.2. Study Design

This study was approved by the ethics committee of the Medical Faculty of the Friedrich-Alexander-Universität Erlangen-Nürnberg (Approval Az:01_2010), and informed consent was obtained from all subjects involved in the study.

To ensure that the selected patient cohort was comparable to the cohort that was used for the originally developed model, we applied the same selection criteria. All patients met the following three criteria: (1) RA patients in sustained remission, defined as a DAS-28 ESR of less than 2.6 for at least 6 months; (2) patients receiving bDMARDs or biosimilars; and (3) time between two included visits was equal to or less than 14 weeks. Patient characteristics were retrieved from medical records. 

**Figure 1:**
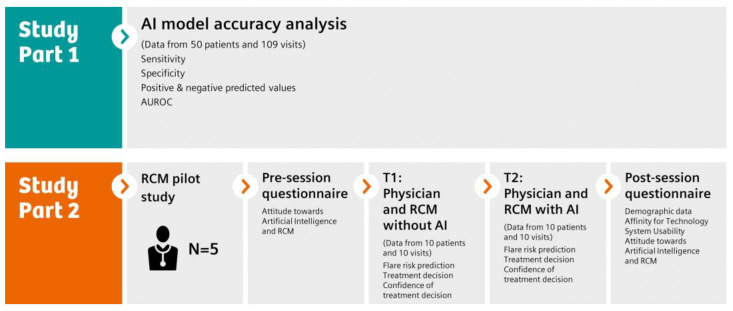
Flow chart of the study.

The study was divided into two main parts (Figure 1), study parts 1 and 2. In study part 1, an AI-powered RA flare risk prediction tool was developed and analysed for accuracy using the data of 50 patients and 109 visits. In study part 2, for a total of 10 case vignettes of real patient cases, *n* = 5 physicians first without access (T1) and then with access to the Rheuma Care Manager (RCM) (T2) provided a flare risk prediction, a treatment decision, and their confidence in the treatment decision. Their attitude towards technology and their user experience were surveyed using various pre- and post-session questionnaires.

### 2.3. Flare Prediction Accuracy

Flare prediction accuracy was estimated as sensitivity, specificity, positive and negative predicted values, and the area under the receiver operating curve (AUROC). An average cohort flare risk of 23% was used as a cut-off as described in other studies [22] to translate continuous flare risk into a binary outcome: Patients with a flare risk < 23% were labelled as “no flare” records and patients with a flare risk ≥ 23% were accordingly labelled as “flare” records. Based on this labelling, we compared the true outcome with the predicted outcome. A confusion matrix was created to visualize the outcomes.

### 2.4. Attitudes towards Technology and AI

Physicians were recruited at the University Hospital Erlangen and were provided a usage guide for the RCM including the flare risk prediction tool. The guide gave an overview about its general structure, functions, and user interface (UI) elements. Attitudes towards technology and AI were surveyed in pre-and post-session questionnaires. Physicians were asked to fill out a baseline questionnaire assessing their personal attitude towards AI and general acceptance of a CDSS in rheumatology using the “Affinity for Technology Interaction (ATI) Scale” developed by Franke, Attig, and Wessel (2019) [23] and the “General Attitudes towards Artificial Intelligence Scale (GAAIS)” developed by Schepman and Rodway (2020) [24]. The physicians were surveyed again with the same questionnaires (ATI and GAAIS) after applying the RCM.

The ATI scale comprises 9 items (e.g., ‘I like to occupy myself in greater detail with technical systems’) and responses are provided on a 6-point response scale ranging from 1 (‘Completely disagree’) to 6 (‘Completely agree’). Negatively worded items were recoded prior to the computation of mean scores across all 9 items, i.e., higher scores represented a higher affinity for technology.

The GAAIS consists of a positive subscale comprising 12 items and an 8-item negative subscale based on a 5-point rating scale from 1 (‘Strongly disagree’) to 5 (‘Strongly agree’). For data analysis, items on the negative subscale were inverted, and individual mean scores were calculated for each subscale separately so that higher scores indicated a more positive attitude towards AI [24].

### 2.5. Comparison of Flare Prediction with and without Access to the Flare Risk Prediction Tool

Flare prediction with and without access to the AI-powered flare risk prediction tool was compared in terms of flare risk, patient features considered most relevant for flare prediction, therapeutic decisions, and confidence.

**Figure 2:**
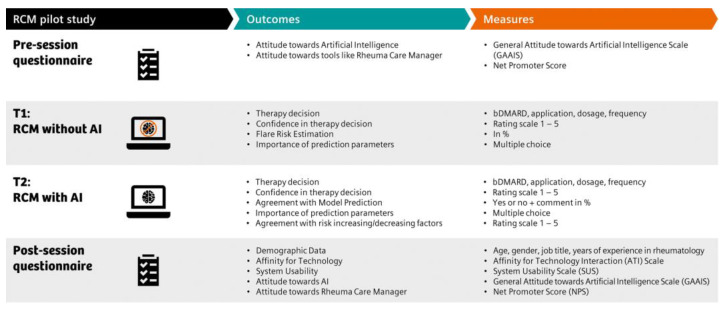
Outcomes and measures of the pilot study. The outcomes (2nd column, petrol) and measures (3rd column, orange) of part 2 are listed in table form and assigned individual study sections (1st column, black) line by line.

At T1, all study participants were presented the data subset of 10 RA patients, with access restricted to the RCM overview feature (T1) and no access to the actual AI-powered flare risk predictions. After studying each case, physicians completed a feedback form to assess their therapeutic decision, perceived flare risk, and confidence in treatment decision, see Figure 2.

During the second part (T2), participants evaluated the same set of 10 patients and completed the same feedback form, now additionally having access to the prediction feature. Physicians completed a questionnaire assessing their affinity for technology, attitude towards AI, perceived system usability, and acceptance of the RCM. In addition, participants could provide feedback on RCM advantages and barriers and provided their basic demographic information and years of professional experience in rheumatology.

#### 2.5.1. Flare Risk Estimation

To provide an individual estimation of a patient’s flare risk at T1, physicians were asked to estimate the risk of a flare within the following 3 months if medication is not adjusted and if medication dosage is cut in half. Responses were given as percentages. Participants had the chance not to answer this question if they felt they could not make an estimation at all. Similarly, at T2 (access to flare prediction), physicians were asked whether they agreed with the predicted flare risk. Again, they answered this question for full and half medication. If they did not agree, they were asked to provide their own estimation in percent.

#### 2.5.2. Patient Features Relevant for Flare Prediction

For each case, participants chose what prediction parameters they considered as most relevant by selecting one or multiple options from the following parameters: (current) dosage, (no) intravenous administration, anti-CCP, BMI, bDMARD, clinical disease activity index (CDAI), CRP, co-therapy, DAS28-ESR, disease duration, ESR, evaluator visual analogue scale (VAS) activity (mm), gender, HAQ, patient VAS activity (mm), patient VAS pain (mm), simple disease activity index (SDAI), SJC, smoker status, and TJC.

#### 2.5.3. Therapeutic Decisions and Confidence

For each patient, participants decided on whether to continue with current medication, change the dosage and/or type of bDMARD, or discontinue treatment with biologics. If they chose to change the dosage, they determined a new dose (in mg) and frequency. If they chose to change the type of bDMARD, they additionally selected the new bDMARD from a comprehensive list and the type of application (oral, subcutaneous, or intravenous). Subsequently, participants rated how confident they felt with their treatment decision on a 5-point scale from 1 (‘not confident at all’) to 5 (‘completely confident’). If physicians decided to change co-therapy, they could leave an open comment to describe. A one-sided paired samples Wilcoxon test was used to assess significance.

### 2.6. Inter-Rater Agreement

Agreement among rheumatologists (raters) was evaluated for a subset of 10 patients with regard to treatment decisions (‘continue’, ‘taper’, or ‘escalate medication’) and the perceived flare risk (in %). Inter-rater reliability scores such as intraclass correlation (interpretation given by Cicchetti (1994) [25]) or Fleiss’ kappa (interpretation given by Landis and Koch (1977) [26]) were not applied in this study since the items were not randomly selected by purpose. Instead, the standard deviation per patient was used as a measure of agreement for the risk estimation. For treatment decision, the number of raters per decision and patient was considered.

### 2.7. Usability and Acceptance

The usability and acceptance of the RCM was measured using the system usability scale (SUS) and the net promoter score (NPS). The SUS is a widely established tool within the field of usability research [27]. Its 10 items (e.g., ‘I think that I would like to use this system frequently’) were answered on a 5-point scale from 1 (‘Strongly disagree’) to 5 (‘Strongly agree’). Individual overall SUS scores were determined following the procedure described by Lewis et al. [28], resulting in scores ranging from 0 to 100 in 2.5-point increments, where scores >68 were considered as above average, scores >80 as high, and 100 representing best possible usability [29]. To interpret individual SUS scores, corresponding adjectives (e.g., ‘good’ or ‘excellent’) identified by Bangor et al. [30] were added.

The NPS, initially introduced by Reichheld [31], provides a summary of consumer satisfaction using a single question. Before using the RCM (T1), a generic description of a rheumatology CDSS was given, and participants were asked ‘How likely are you to recommend such a tool to other colleagues?’ and responded on a 11-point scale ranging from 0 (‘Very unlikely’) to 10 (‘Very likely’). Based on their ratings, individuals were considered either ‘promoters’ (rating 9 or 10), ‘passively satisfied’ (rating 7 or 8), or ‘detractors (rating 0–6) of the product. To calculate an overall NPS, the percentage of detractors was subtracted from the percentage of promoters [31]. After using the RCM (T2), the same question was asked. Mean rating scores were also calculated.

## 3. Results

### 3.1. Flare Prediction Accuracy

**Figure 3:**
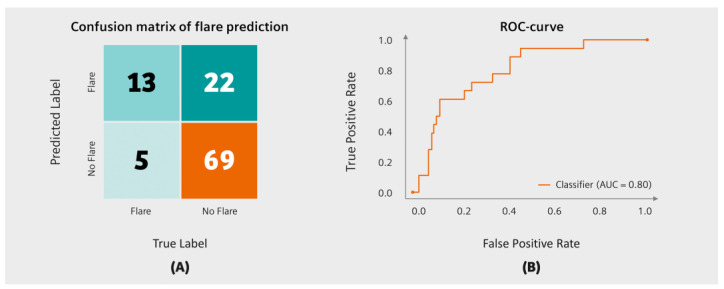
Flare prediction accuracy. (**A**) Confusion matrix of flare prediction and (**B**) receiver operating characteristic (ROC) curve.

Data of 50 RA patients (Table 1, Table 2 and Table 3) with a total of 109 recorded visits from the University Clinic Erlangen were used to assess model accuracy. The tool predicted RA disease flares with a sensitivity of 72% (95% CI, 31–85%), a specificity of 76% (95% CI, 68–84%), a positive predicted value of 37% (95% CI, 13–52%), a negative predicted value of 93% (95% CI, 79–98%), and an AUROC of 80% (95% CI, 53–86%), see Figure 3. The total accuracy of the flare risk prediction tool (equal to the number of correctly predicted events divided by the total number of predictions) was 75% (95% CI, 71–89%).

### 3.2. Pilot Study

Five physicians (three female) with a mean age of 29.4 years working at the rheumatology outpatient clinic of the University Clinic Erlangen with varying years of work experience in rheumatology (1–5 years) were included. Four residents in training with 1, 2, 3, and 5 years of training and one board-certified rheumatologist with 5 years of training also participated in the study.

#### 3.2.1. Technology and AI Affinity

The average affinity for technology was 4.13 (SD = 0.41) and general attitudes towards AI improved slightly after using the RCM, from 4.10 (SD = 0.53) to 4.17 (SD = 0.67) for the positive subscale of the GAAIS and from 3.65 (SD = 0.38) to 3.88 (SD = 0.41) for the negative subscale. A trend indicated that participants with a higher affinity for technology had a more positive attitude towards AI after using the system compared to participants with a relatively low affinity for technology, Table 4.

#### 3.2.2. Flare Risk Prediction

**Figure 4:**
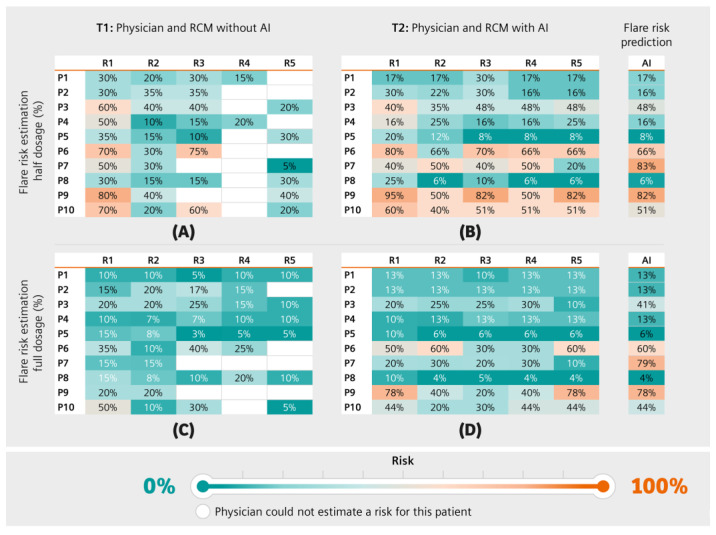
Flare risk prediction. Physicians predicted disease flare risks for half dosage (**A**,**B**) and full dosage (**C**,**D**) at T1 (before flare risk prediction tool usage, **A**,**C**) and at T2 (after prediction tool usage, **B**,**D**). AI represents the AI-based flare prediction probability (single column table on the right). Petrol represents low flare risk and orange indicates high flare risk. White denotes that no assessment could be made.

The physicians predicted varying disease flare risks (Figure 4), agreeing with 54% and 52% of the AI-based predicted flare risk in patients for full and half medication doses, respectively. The lowest agreement was found in T1 for the estimation of the full dosage risk, with an average standard deviation per patient for risk estimation of 16%. For T1, the average standard deviation per patient for both the half and full dosage was 6%, whereas in T2 it was 8% for half dosage and 7% for full dosage, indicating a moderate agreement.

**Figure 5:**
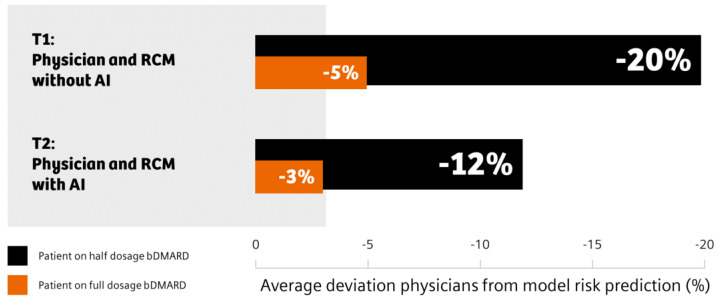
Deviation of flare risk predicted by physicians compared to the flare risk prediction tool. Mean flare risk deviations in percent between physicians and the AI-powered flare risk prediction tool at T1 (physicians had no access to the flare prediction tool, upper bar graphs) and at T2 (physicians had access to the flare prediction tool, lower bar graphs) are shown. Black represents the flare risk prediction deviation for the patient on a half dosage of bDMARD and orange indicates that for the patient on a full dosage of bDMARD.

Physicians generally reported a lower flare risk compared to the flare risk prediction tool and deviation was higher for the half dosage prediction (Figure 5). The deviation between physicians and the model decreased when physicians were given access to the prediction feature (T2).

**Figure 6:**
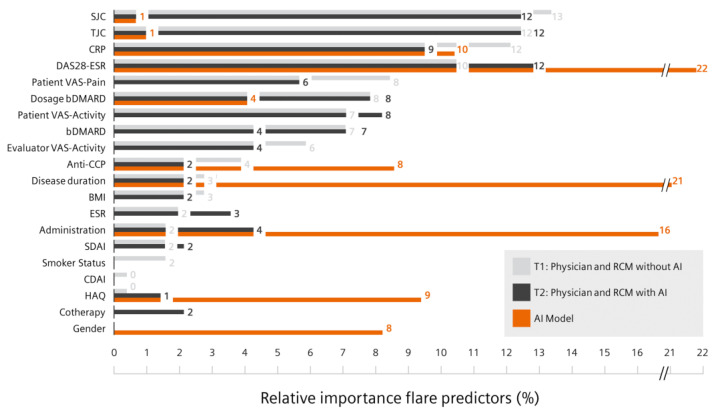
Relative importance of flare prediction parameters for physicians and the flare prediction tool. Relative importance of single flare predictors (*y*-axis) for the physicians as mean value at T1 (grey) and T2 (black) and for the flare prediction tool (orange) shown in percent (*x*-axis). SJC—swollen joint count; TJC—tender joint count; CRP—C-reactive protein; DAS28—disease activity score 28 joints; ESR—erythrocyte sedimentation rate; VAS—visual analogue scale; bDMARD—biologic disease-modifying anti-rheumatic drugs; anti-CCP—anti-cyclic citrullinated peptide; BMI—body mass index; SDAI—simple disease activity index; CDAI—clinical disease activity index; HAQ—health assessment questionnaire.

The physicians rated swollen and tender joint count as the most important features, whereas DAS-28-ESR and disease duration were the most important features for the AI-powered flare risk prediction tool (Figure 6). Supplementary Figure S1 and Figure S2 display all physician feature ratings. HAQ and gender were not considered relevant by physicians, compared to 9% for the flare risk prediction tool.

#### 3.2.3. Treatment Decisions and Perceived Confidence

**Figure 7:**
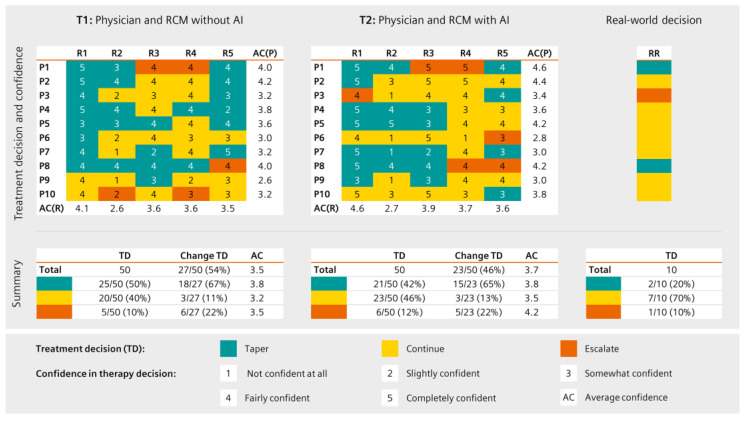
Treatment decisions of physicians (R1–R5) for the ten patient vignettes (P1–P10) according to the five physicians and the real-world treatment decision (RR). The type of decision is shown in different colours (tapering—petrol; no change—yellow; escalation—orange). Perceived confidence in treatment decision per patient and physician and average confidence are also displayed.

The treatment decisions were heterogenous (Figure 7), and all physicians made the same treatment decision (taper, continue, or escalate) in none of the cases (T1 and T2). Physician agreement was poor, yet increased slightly when physicians had access to the prediction features (T2); RCM usage led to more tapering decisions (T1:50%; T2:42%) compared to the original decisions by the treating physicians (20%). At T1 (no access to the flare prediction tool) 27/50 (54%) and at T2 (access to the flare prediction tool) 23/50 (46%) treatment category changes were observed compared to the original decisions.

Similarly, confidence in treatment decisions was heterogenous regarding the different patients and participating physicians (Figure 7). At T2, a numerical (*p* = 0.052) mean confidence increase from 3.5 (SD = 0.95) to 3.7 (SD = 1.20) was observed. Mean confidence in escalation increased from 3.5 to 4.2 such that at T2, continuing current treatment was the decision with the least mean confidence.

#### 3.2.4. RCM Usability and Acceptance

Usability was rated good with a mean SUS score of 82/100. The NPS decreased from +40% to −20% after usage. The mean ratings were 8/10 at T1 and 7/10 at T2, indicating a passive acceptance of the tool. 

#### 3.2.5. Perceived RCM Advantages and Barriers

Physicians generally reported positive impressions of the RCM (see Table 5). They especially valued the feature that provided patient-specific personalized information, which could be used to support tapering decisions. They mentioned concerns regarding the limited amount of patient information currently available in the system, the risk of potential over-reliance on the system, and the difficulty to engage patients. The visualization of patient data on the overview page was perceived as helpful by most physicians, and even favourable in comparison to conventional systems.

## 4. Discussion

In the present study, the accuracy of a novel CDSS, called RCM, including an AI-powered flare prediction tool was investigated. Additionally, the usability, acceptance, and potential influence of RCM on physician decision making were explored. CDSSs have already been found to be helpful for the diagnosis of RA [32,33], and the validity of flare prediction applications has been previously shown for RA and giant cell arteritis. Results from ongoing longitudinal studies testing the benefit of such tools are still lacking [34]. To our knowledge, our study is the first to test the usability and acceptance of a flare prediction tool by physicians using real-world patient case vignettes.

One of our objectives was to evaluate the prognostic quality of our model based on unknown data. Overall, the accuracy, sensitivity, and specificity of our flare prediction tool were promising and roughly in the range of those reported in previous studies [18,35,36,37,38]. The heterogeneity of physician predictions and the large discrepancies between the flare prediction tool and physician estimates were two important and simultaneously alarming findings. Only case vignettes of patients in remission were included in the study, which may have contributed to the heterogeneity of the assessments as T2T tapering recommendations are less clearly defined compared with dose escalation strategies in the current ACR and EULAR guidelines [4,5]. In fact, there is generally disagreement about which patients are particularly suitable for tapering, which became evident from a comparison of larger observational studies showing large between-country differences in patient characteristics such as age and comorbidities [39,40,41].

The degree of heterogeneity in assessments between physicians in this study may suggest the usefulness of standardizing decision aids. The decrease in the deviation between physicians and the prediction tool when physicians were given access to the prediction feature was a promising observation in this respect.

Physician treatment confidence greatly varied depending on patient cases and in general. Although, to some extent, this uncertainty could also be due to the unfamiliarity of dealing with case vignettes without the possibility of interviewing and clinically examining the real patient, we again see evidence here for the need and usefulness of a CDSS to support therapy decisions. Despite the small group size, a clear trend toward increased confidence in treatment decision making was demonstrated with access to the prediction feature, highlighting another benefit of the application. This aspect was further underlined by the qualitative analysis in this study, where several users emphasized the clarity of the tool and the feeling of security when assessments were consistent. The basically positive attitude towards AI and technologies tended to even increase when trying out the new flare prediction tool.

Therapeutic decisions, in which many aspects must be included and integrated at once, can be very difficult and are therefore prone to bias [42]. Stress and time pressure, which frequently affect physicians, further complicates the decision-making process [43]. All parameters that were included in the flare prediction feature can be collected without much time and effort. Despite its supposed predictive value [44,45], the inclusion of imaging criteria was specifically omitted for this reason. This pragmatic approach sets our tool apart from other more sophisticated ones [46,47,48] and facilitates its direct use in clinical practice.

Using the flare prediction tool increased therapy changes in particular with respect to tapering decisions, and the majority of RCM users tapered in more patients than the treating physician. This trend toward more tapering is consistent with current developments in RA, where more and more patients are in sustained remission, for which tapering has been shown to be feasible, and an increasing cost pressure from biologicals [49,50,51].

The relative importance of flare prediction parameters differed between raters and the flare risk prediction tool. While the RCM heavily weighted DAS-28 ESR, disease duration, route of application of the biological, anti-CCP status, gender, and HAQ, raters were more likely to include the individual components of the DAS-28 (SJC and TJC) and patient VAS in their decision. The selection of parameters for the AI-powered risk prediction was justified by their relative importance with respect to flare prediction. The selection of the raters, however, was probably based more on experience and intuition. HAQ and gender were not included in the rater’s decision making, although previous studies have shown predictive and prognostic value for these parameters [52,53,54]. Although the exact reasons for this remain elusive, e.g., information overload and practical reasons such as the unavailability of scores collected on paper in the decision-making situation, seem plausible [55].

This study has some limitations. The effects achieved by the RCM appear quite small, reflecting the small sample of five relatively young physicians. Subsequent larger studies and ultimately a comparative study where the RCM is compared to the standard of care in real patients are needed. Moreover, the RCM was evaluated without including patients as the most important co-decision makers, possibly jeopardizing shared decision making. However, supporting the decision with a CDSS could also give patients, who are conflicted between drug and disease burden, confidence to participate in treatment decisions. Furthermore, we cannot eliminate the possibility that presenting an AI-predicted flare risk reduced the variability between physician’s judgments via an anchoring bias, in that they relied too heavily on the proposed value in their decision making. However, given the attempted standardization of decision making through the RCM, such an approximation is desirable under the assumption that the AI-based prediction is accurate. The qualitative analysis revealed physician concerns about the reliability of the tool and the slight decrease in the NPS after usage expressed scepticism. It is important to note that the physicians did not know the prediction tool’s accuracy when they provided their evaluations. A manual that explains the most important facts, including flare-prediction accuracy, could promote usage and trust. Concerns were expressed that the use of the tool in clinical practice could trigger uncertainty in the case of deviating assessments. However, disagreement between the physician and the prediction tool could also induce further reflection on the patient’s case and consultation with colleagues, which would ultimately have a positive impact on patient care.

## 5. Conclusions

In conclusion, the AI-based RCM yielded promising results regarding validity, usability, and acceptance. We are now planning further longitudinal studies in larger cohorts to test its use in real clinical practice and explore patient acceptance.

## 6. Patents

EP21165619.4, CN202210301121.4, US17/703,226; EP21182428.9, CN202210733378.7, US17/848,993.

## Figures and Tables

**Table 1:** Baseline patient characteristics for model quality assessment. Values are means (SD) if not stated otherwise. Only particular visits and 10 variables were considered for assessing model quality. **SJC**—swollen joint count; **TJC**—tender joint count; **CRP**—C-reactive protein; **DAS–28**—disease activity score 28 joints; **ESR**—erythrocyte sedimentation rate; **bDMARD**—biologic disease-modifying anti-rheumatic drugs; **anti-CCP**—anti-cyclic citrullinated peptide; **BMI**—body mass index; **HAQ**—health assessment questionnaire.

Patient Characteristics Study Part 1 (*n* = 50)	
DAS-28 ESR, units	1.32 (0.61)
Disease duration, years	11.34 (9.61)
IV administration, N (%)	38 (34.9)
Anti-CCP positive, N (%)	73 (66.9)
Female gender, N (%)	65 (59.6)
HAQ, mean score	0.38 (0.8)
CRP, mg/dL	0.3 (0.78)
Full dosage bDMARD, visits (%)	334 (70.5)
SJC, N	0.2 (0.66)
TJC, N	0.17 (0.48)

**Table 2:** Characteristics of the 10 patients included in part 2. Values were aggregated over the whole visit history if not stated otherwise. Smoker status was only available for 9 out of 10 patients. **CRP**—C-reactive protein; **DAS28**—disease activity score 28 joints; **TJC**—tender joint count; **HAQ**—health assessment questionnaire; **SJC**—swollen joint count; **VAS**—visual analytics scale; **SDAI**—simple disease activity index; **CDAI**—clinical disease activity index; **csDMARD**—conventional synthetic disease-modifying anti-rheumatic drugs.

Patient Characteristics Study Part 2 (*n* = 10)	
Age, years	57.7 (6.2)
Female gender, N (%)	7 (70)
Disease duration, years	15.7 (10.8)
Smoking, N (%)	
Current smoker	4 (40)
Ex-smoker	2 (20)
Never smoker	3 (30)
Remission duration, months	58.3 (7.6)
DAS-28 ESR, units	1.5 (0.6)
TJC, N	0.65 (0.81)
SJC, N	0.36 (0.44)
CRP, mg/dL	4.8 (4.1)
Patient VAS activity (mm)	12.6 (7.35)
IV administration, N (%)	7 (70)
Evaluator VAS activity (mm)	7.3 (5.4)
ESR, mm/h	6.2 (3.5)
(Current) anti-CCP positive, N (%)	8 (80)
BMI, kg/m²	27.8 (6.9)
SDAI, units	7.8 (4.7)
HAQ, units	0.9 (0.8)
CDAI, units	2.7 (2)
Methotrexate use, N (%)	4 (40)
Other csDMARD use, N (%)	3 (30)
bDMARD use, N (%)	10 (100)
Adalimumab	2 (20)
Tocilizumab	5 (50)
Certolizumab pegol	1 (10)
Rituximab	2 (20)
(Current) dosage, %	80 (27.4)
Patients with flare, N (%)	3 (30)

**Table 3:** Patient characteristics of the 10 patients included in part 2. CRP—C-reactive protein; DAS28—disease activity score 28 joints; TJC—tender joint count; HAQ—health assessment questionnaire; SJC—swollen joint count.

Patient (P)	Characteristics (Reason for Selection)
P1	Low disease duration
P2	High CRP
P3	High CRP
P4	High TJC
P5	High disease duration, low DAS28
P6	High HAQ
P7	High disease duration, high DAS28
P8	At least one TJC and SJC
P9	Random
P10	Random

**Table 4:** GAAIS, ATI, and NPS scores of physicians according to respective study phase. GAAIS—general attitudes towards artificial intelligence scale; ATI—affinity for technology interaction (ATI) scale; NPS—net promoter score; SUS—system usability scale; SD—standard deviation.

Rater (R)	GAAIS				NPS		ATI	SUS
	Positive Subscale		Negative Subscale					
	Pre-Study	Post-Study	Pre-Study	Post-Study	Pre-Study	Post-Study		
R1	4.75	5.00	3.88	4.25	9	10	4.33	100.0
R2	4.08	4.25	3.13	3.63	7	6	4.22	80.0
R3	4.50	4.58	3.63	3.50	7	7	4.67	92.5
R4	3.50	3.67	4.13	4.38	9	7	3.78	75.0
R5	3.67	3.33	3.50	3.63	8	5	3.67	62.5
**Mean**	**4.10**	**4.17**	**3.65**	**3.88**	**8**	**7**	**4.13**	**82**
SD	0.53	0.67	0.38	0.41	1	1.87	0.41	14.73

**Table 5:** Perceived RCM advantages and barriers.

Advantages Mentioned	Problems Mentioned
Decision support for when and how to taper Feedback on individual risk for patients (instead of standard populations); (*“This helps immensely to implement shared decision-making.”*—R1) Possibility to integrate more data to make the tool even more helpful Increased feeling of confidence, especially when there was a large agreement between rater and model prediction Clear overview over patient’s history of therapies and disease activity (*“This allows for faster decision-making. Data visualization is in general much better with this tool.”*—R3)	Sometimes lack of agreement between model flare risk and risk predictors (*“This can generate insecurity in the user. Users should be taught to interpret and contextualize this function of the tool.”*—R3) Limited amount of clinical data (e.g., radiological results, comorbidities, or data on infections) Concern that physicians could rely too heavily on the model prediction while ignoring other patient data Potential risk of more time needed if prediction values were discussed with patients Partly unclear visualization (*“I find it difficult to distinguish between the real risk and the average risk of flare.”*—R5)

## Data Availability

Data are available upon request.

## Supplementary Materials

The following supporting information can be downloaded at: https://www.mdpi.com/article/10.3390/diagnostics13010148/s1, Figure S1: Feature importance rating of physicians (T1), Figure S2: Feature importance rating of physicians (T2).
