# Supplementary material for: An AI-Powered Clinical Decision Support System to Predict Flares in Rheumatoid Arthritis: A Pilot Study
Source: Diagnostics (Basel). 2023 Jan 1;13(1):148. doi: 10.3390/diagnostics13010148 (PMC9818406; doi:10.3390/diagnostics13010148)
Supplement: Supplementary file 1 [file diagnostics-13-00148-s001.zip › diagnostics-2012723-supplementary.pdf]

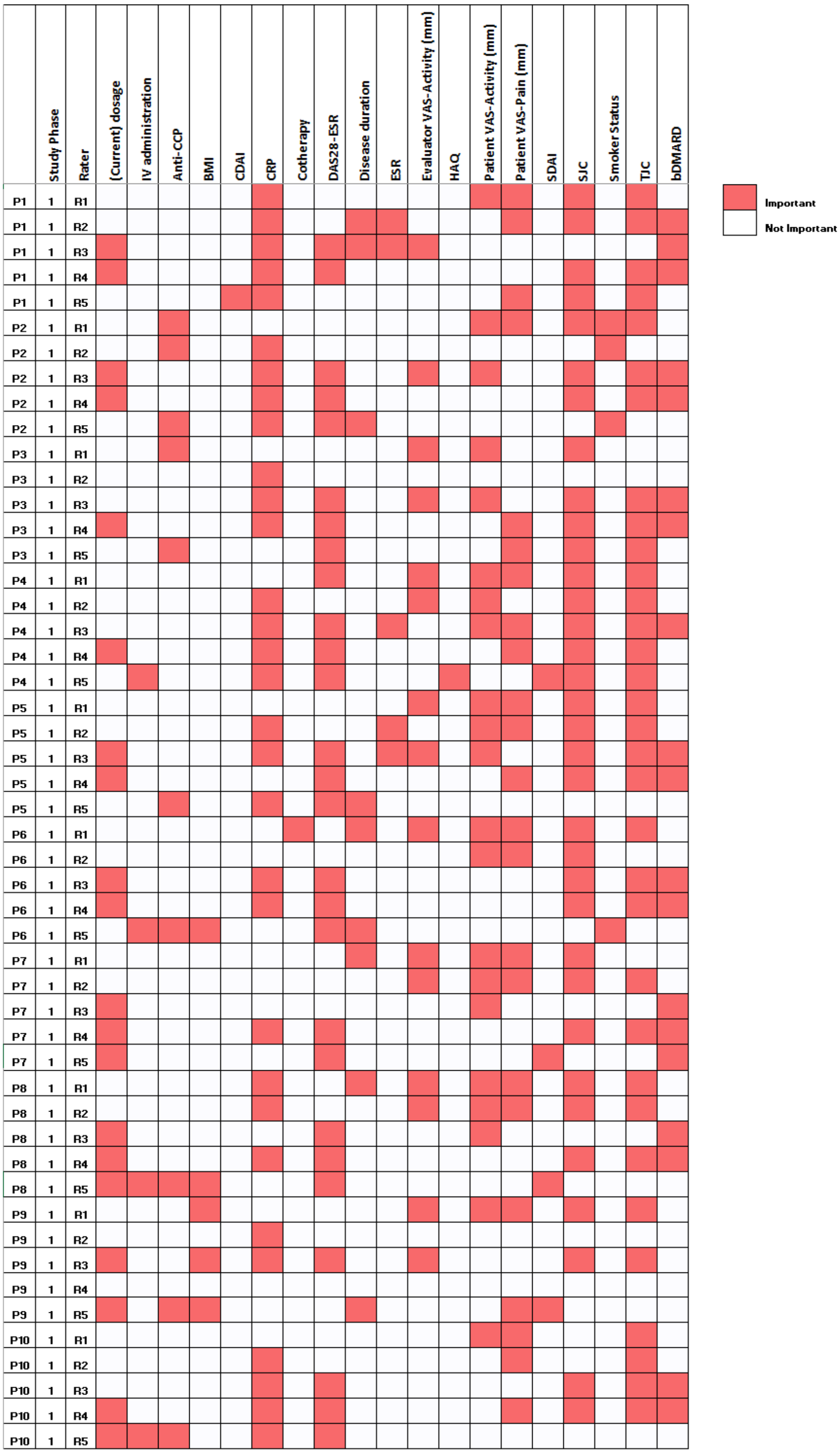

Figure S1: Feature importance rating according to individual patient and physician (T1).

IV, intravenous; Anti-CCP, anti-citrullinated protein antibody; BMI, body mass index; CDAI, Clinical Disease Activity Index; CRP, C-reactive protein, DAS28-ESR, Disease Activity Index 28-erythrocyte sedimentation rate; VAS, visual analogue scale; HAQ, Health Assessment Questionnaire; SDAI, Simple Disease Activity Index; SJC, swollen joint count; TJC, tender joint count; bDMARD, biologic Disease-Modifying Antirheumatic Drugs. Red represents that the parameter was rated as important, white as not important.

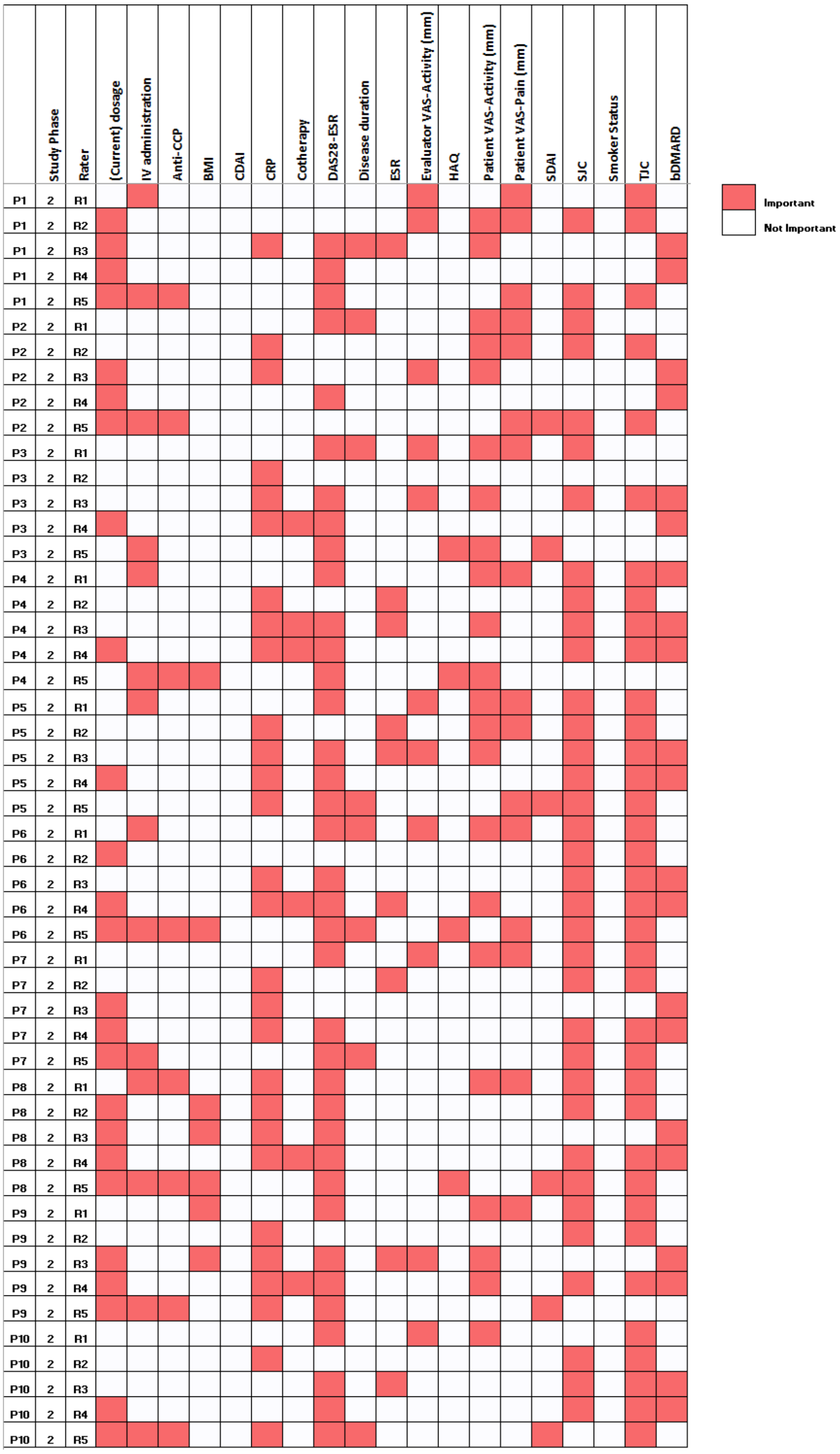

Figure S2: Feature importance rating according to individual patient and physician (T2).
IV, intravenous; Anti-CCP, anti-citrullinated protein antibody; BMI, body mass index; CDAI, Clinical Disease Activity Index; CRP, C-reactive protein, DAS28-ESR, Disease Activity Index 28-erythrocyte sedimentation rate; VAS, visual analogue scale; HAQ, Health Assessment Questionnaire; SDAI, Simple Disease Activity Index; SJC, swollen joint count; TJC, tender joint count; bDMARD, biologic Disease-Modifying Antirheumatic Drugs. Red represents that the parameter was rated as important, white as not important.
